# Evaluating the impact of the Health Access for Refugees Project on people who are refugees or seeking asylum in Northern England

**DOI:** 10.1177/17579139241270768

**Published:** 2024-10-14

**Authors:** M-C Balaam, M Haith-Cooper, D Mathew

**Affiliations:** School of Nursing and Midwifery, University of Central Lancashire, Preston PR1 2HE, UK; Faculty of Health Studies, University of Bradford, UK; HARP Volunteer, Leeds Refugee Council, UK

**Keywords:** asylum seekers and refugees, health care barriers, health care access, public health intervention

## Abstract

**Aims:**

Evidence demonstrates that people who are seeking asylum and refugees face individual, institutional and system-level barriers when accessing health services. Health Access for Refugees’ Project (HARP) is a UK initiative increasing access to health care within this community through a series of interventions. This study explored the impact of HARP on health service access, experiences and outcomes for clients, and how volunteers and staff addressed institutional and system-level barriers.

**Methods:**

In summer 2020, we conducted qualitative telephone interviews with four HARP clients, eight clients who became volunteers, seven further volunteers and three staff members.

**Results:**

The educational aspect of the interventions supported clients navigating the complex UK health care system while promoting independence in accessing health care. Advocacy by volunteers and staff was important in challenging barriers at individual and institutional levels. Staff challenged the asylum system, by improving information around entitlement to health care and addressing barriers to registering with a General Practitioner (GP).

**Conclusions:**

Interventions such as those provided by HARP can address different levels of barriers to support people accessing health care provision. This can be achieved through training health professionals and working with peers to support access to care and to develop self-advocacy. However, stable long-term funding is essential to ensure the sustainability of these initiative.

## Background

Asylum seekers and refugees (AS&R) have disproportionately poor physical and mental health compared to the general population.^1^ They may experience insecure housing, precarious legal status, and lack of employment and education, which can exacerbate poor health.^2^ They face barriers accessing health services, including language, culture, social deprivation health literacy, and non-eligibility for care.^3^ The Health Access for Refugees Project (HARP) delivered by the Refugee Council aimed to improve access to healthcare with peers trained to co-deliver interventions. A full evaluation of the project was undertaken.^4^ This article presents findings on the impact of HARP on healthcare access.

## Methods

Following ethical approval HARP staff discussed the study with potential participants on the phone or online. Between June and August 2020, 22 audio-recorded telephone semi-structured interviews were undertaken with AS&R clients (C; *n* = 4), clients who became volunteers (VC; *n* = 8), volunteers (V; *n* = 7) and staff (S; *n* = 3). Participants were purposefully selected, using sample stratification to ensure balance of gender, age and country of origin. Data were anonymised and transcribed verbatim, and then thematically analysed.^5^ Full details of the evaluation are available (ref).

## Results

See Table 1 for participants’ demographics.

**Table 1.** Participants’ demographics

Code	Role	Country of origin	Status	Arrived in the United Kingdom
1_C	Client	Iraq	Asylum seeker	2019
2_C	Client	Sudan	Asylum seeker	2015
3_C	Client	Syria	Refugee	2019
4_C	Client	Lebanon	Asylum seeker	2019
1_VC	Volunteer/client	Pakistan	Asylum seeker	2015
2_VC	Volunteer/client	Iran	Asylum seeker	2020
3_VC	Volunteer /client	Eritrea	Refugee	2019
4_VC	Volunteer/client	Albania	Asylum seeker	2019
5_VC	Volunteer/client	El Salvador	Asylum seeker	2019
6_VC	Volunteer/ client	El Salvador	Asylum seeker	2019
7_VC	Volunteer/client	Iraq	Asylum seeker	2019
8_VC	Volunteer/client	Zimbabwe	Asylum seeker	2017
1_V	Volunteer	Albania	Asylum seeker	2013
2_V	Volunteer	Albania	Asylum seeker	2015
3_V	Volunteer	Pakistan	Asylum seeker	2018
4_V	Volunteer	Iraq	Asylum seeker	2002
5_V	Volunteer	Egypt	Asylum seeker	2012
6_V	Volunteer	Albania	Asylum seeker	2018
7_V	Volunteer	Iraq	Asylum seeker	2008
1_S	Staff	N/A	N/A	N/A
2_S	Staff	N/A	N/A	N/A
3_S	Staff	N/A	N/A	N/A

Four themes emerged.

### Education for AS&R and health staff to ensure access to care

Newly arrived AS&R discussed how HARP helped them to understand the UK healthcare system. Volunteers and staff directed clients to services, including dentists, opticians, maternity care, and General Practitioners (GPs). One participant described how HARP helped when she was charged for prescriptions:

*The first time she bought medicine she paid for it because she didn’t know the HC2 form is to get medicine for free. And after that she knows now.* (4_VC)

HARP also educated health professionals such as local GPs about the healthcare needs of AS&R people. One GP trainer cascaded her knowledge to other GPs:

*[The GP trainer] came and shadowed me and the volunteers all day . . . she spoke to clients and asked the problems they were facing with GPs and receptionists. And she then came along to our ESOL for Health English class. . .. And she got the opportunity to explain who she was and ask for feedback, and she ended up in tears within that session . . . and she designed a presentation about the barriers that asylum seekers are facing. And she delivered the training to, I think. . .she said something like it was seventy or eighty trainee GPs across the Yorkshire area.* (3_S)

### Providing advocacy for better healthcare

Several clients described how HARP volunteers acted as advocates, supporting them in accessing healthcare and wider services:

*I am disabled and they just ignoring me, I went to there and I gave them doctor letter and they didn’t do anything for me. But since I talk to [HARP member] she find out and straightaway I’ve got a bus ticket, you know, like free ticket bus*. (1_C)

Advocacy led to improved health outcomes, for example, a pregnant diabetic woman did not attend antenatal appointments after being inappropriately charged for care. HARP’s support led to her bills being cancelled facilitating access to the care she needed:

*I was scared, and I was, I thought it must, I’m no longer going to the hospital because if I keep going they’ll keep sending bills.* (2_C)

Volunteers acted as interpreters when clients experienced language barriers and advocated for interpreters when services did not provide them:

*[we] emailed them from the Refugee Council, from HARP saying that what had happened, and what, asking why hadn’t they used an interpreter because this is a human right to be understood. . .I got an email back from the dentist saying, oh there’d been a mistake, the receptionist didn’t know, and please reassure him that we will use interpreters in the future, and that they’d be booked, and now he is getting interpreters.* (1_S)

### Building confidence to access and navigate healthcare services

Through HARP, clients developed the confidence to access healthcare independently but were reassured that HARP could step in if they struggled in the future:

*now she feels confident to go to the GP, opticians and dentist but not so much to go to the hospital, she feels like the hospital is too big for her and she can’t manage properly.* (4_VC)

However, some clients who had complex needs requiring multiple services, or those with limited English believed they would need ongoing support:

*He said that even in the future, he thinks he will need [HARP worker] always to interfere because he said that things have complicated and we don’t know most of it.* (3_C)

HARP helped clients build the confidence to speak out when care was substandard. One woman experienced language barriers when visiting her GP and was facilitated to complain about this care:

*She got told some information, she didn’t understand it at that time. . . she was trying to explain, in the very best English she could, while trying to show her phone . . . the receptionist was just getting irritated, . . .and just blurting things back to her, . . .She started crying at reception, you know. So she attended the patient panel meeting with me, and she told the practice manager, the receptionist, she told the other patients that were on the panel exactly what had happened to her. . .. Because of this, different things got put in place. we got the receptionists there to start wearing name badges. . .they were more aware of clients that were new to this country and had got a language barrier, they became more patient.* (3_S)

### Challenging barriers to access statutory systems

HARP staff identified and challenged institutional- and system-level barriers affecting access to healthcare, for example, when housing providers did not issue exemption forms for services in a timely manner:

*they’re not acquiring HC2s for the people that are in there* (hotels), *and I had a meeting . . .where I was reiterating, if you don’t issue HC2s for people, they will get charged if they go to the dentist, they will get a fine*. (2_S)

At a national level, HARP challenged the quality of written information for asylum seekers accessing a GP:

*I’ve been involved with national meetings about this with Doctors of the World, and NHS England, and different groups, reviewing the leaflets.* (1_S)

This led to national policies being changed to ensure AS&R people can act as patient representatives on national committees:

*there could be at least 80 odd at a meeting. . .not one person there had been through the* (asylum) *system themselves. . .I’ve challenged them on the NHS logo, slogan is, ‘No decision about me without me’, and I think well why are we white middle class people deciding what should happen.* (1_S)

## Discussion

This study presents aspects of an evaluation of the HARP project, focusing on healthcare access, and we found that HARP interventions facilitated access to healthcare. These included educating clients about available services and HARP staff challenging substandard care and systemic barriers at a national level. HARP increased clients’ confidence to access services independently, especially through learning English, while providing a safety net when clients faced difficult barriers. HARP included all the components of a recent systematic review^6^ which reported that barriers to health services in refugee populations could be overcome by involving peers in intervention delivery, ensuring language support through interpreters or peers and delivering education sessions to increase knowledge of services.

Interventions involving peers are increasingly used in public health programmes.^7^ Peers sharing characteristics, including language, cultural, ethnic backgrounds and lived experience with the recipient, can increase the effectiveness of interventions by addressing power imbalances and forming trusting relationships.^8^ This is particularly important for people who may have experienced trauma through the process of claiming asylum.

Advocacy was an important intervention with peer’s advocacy not only facilitating practical support in AS&Rs daily lives but also role modelling to promote self-advocacy. Nurturing self-advocacy through education and support facilitates individuals to become experts by experience, building skills and expertise which will in turn support integration into work and education.^9^ Advocacy also challenged institutional barriers in primary care and hospitals, and organisations such as the UK Home Office and housing companies, which are responsible for resources and policies around healthcare access for AS&R.^2^

Research has found health professionals lack awareness of barriers AS&R face accessing health services^4^ which HARP addressed through educating health professionals. Encouraging health professionals to shadow HARP then educate their peers in clinical practice could be adopted as a model to increase knowledge. Reflecting previous studies,^10^ the education of clients was also a focus of the interventions to overcome barriers, teaching the English language and how to access health services. However, more research is needed to examine how this education influences health outcomes.

## Conclusion

UK-based AS&R face multiple barriers to accessing health services which interventions such as HARP can address. Health professionals need training to ensure they understand the needs of AS&R and the institutional/systemic barriers and discriminatory practices that provide the context to their healthcare experiences. Developing the capacity of AS&R and harnessing peers is essential to reflect the needs of AS&R, while promoting independence and self-advocacy. However, sustainable interventions are important, needing long-term funding by public bodies and health services.
